# Inflammatory Biomarkers of Traumatic Brain Injury

**DOI:** 10.3390/ph15060660

**Published:** 2022-05-25

**Authors:** Nathan H. Johnson, Roey Hadad, Ruby Rose Taylor, Javier Rodríguez Pilar, Osman Salazar, Juan Antonio Llompart-Pou, W. Dalton Dietrich, Robert W. Keane, Jon Pérez-Bárcena, Juan Pablo de Rivero Vaccari

**Affiliations:** 1 Department of Physiology and Biophysics, University of Miami Miller School of Medicine, Miami, FL 33136, USA; nhj11@med.miami.edu (N.H.J.); rxh578@med.miami.edu (R.H.); rkeane@miami.edu (R.W.K.); 2 Department of Neurological Surgery and The Miami Project to Cure Paralysis, University of Miami Miller School of Medicine, Miami, FL 33136, USA; ruby.taylor@med.miami.edu (R.R.T.); ddietrich@med.miami.edu (W.D.D.); 3 Intensive Care Department, Son Espases University Hospital, 07120 Palma de Mallorca, Spain; javier.rodriguez@ssib.es (J.R.P.); osman.salazar@ssib.es (O.S.); juanantonio.llompart@ssib.es (J.A.L.-P.); juan.perez@ssib.es (J.P.-B.)

**Keywords:** inflammasome, inflammation, brain injury, biomarkers, cytokines, interleukin

## Abstract

Traumatic brain injury (TBI) has a complex pathology in which the initial injury releases damage associated proteins that exacerbate the neuroinflammatory response during the chronic secondary injury period. One of the major pathological players in the inflammatory response after TBI is the inflammasome. Increased levels of inflammasome proteins during the acute phase after TBI are associated with worse functional outcomes. Previous studies reveal that the level of inflammasome proteins in biological fluids may be used as promising new biomarkers for the determination of TBI functional outcomes. In this study, we provide further evidence that inflammatory cytokines and inflammasome proteins in serum may be used to determine injury severity and predict pathological outcomes. In this study, we analyzed blood serum from TBI patients and respective controls utilizing Simple Plex inflammasome and V-PLEX inflammatory cytokine assays. We performed statistical analyses to determine which proteins were significantly elevated in TBI individuals. The receiver operating characteristics (ROC) were determined to obtain the area under the curve (AUC) to establish the potential fit as a biomarker. Potential biomarkers were then compared to documented patient Glasgow coma scale scores via a correlation matrix and a multivariate linear regression to determine how respective biomarkers are related to the injury severity and pathological outcome. Inflammasome proteins and inflammatory cytokines were elevated after TBI, and the apoptosis-associated speck like protein containing a caspase recruitment domain (ASC), interleukin (IL)-18, tumor necrosis factor (TNF)-α, IL-4 and IL-6 were the most reliable biomarkers. Additionally, levels of these proteins were correlated with known clinical indicators of pathological outcome, such as the Glasgow coma scale (GCS). Our results show that inflammatory cytokines and inflammasome proteins are promising biomarkers for determining pathological outcomes after TBI. Additionally, levels of biomarkers could potentially be utilized to determine a patient’s injury severity and subsequent pathological outcome. These findings show that inflammation-associated proteins in the blood are reliable biomarkers of injury severity that can also be used to assess the functional outcomes of TBI patients.

## 1. Introduction

Traumatic brain injury (TBI) has a complex and chronic pathology that represents a significant public health concern in the United States and throughout the world [1,2,3,4,5]. It is estimated that in the United States there are 3.17 million people suffering with long term disability resulting from TBI, representing an annual economic impact in excess of $56 billion [6]. TBI presents as a biphasic pathology in which the effects of the initial traumatic insult results in persistent inflammation and the chronic activation of the innate immune system [7,8,9,10]. Primary injury involves the release of damage associated molecular patterns (DAMPS) from injured tissue resulting in the activation of the innate immune response and formation of the inflammasome [11,12,13,14]. Although the levels of DAMPs and PAMPs have been shown to gradually decrease over the first week after injury, chronic inflammatory activity is often seen months to years after injury, resulting in a secondary injury from chronically activated microglia and their subsequent release of inflammatory cytokines [15,16,17].

The inflammasome is a multi-protein complex that activates caspase-1 leading to the cleavage and release of the inflammatory cytokines interleukin (IL)-1β and IL-1, and the formation of a gasdermin-D pore as part of the programmed cell death mechanism of pyroptosis [18,19,20,21,22,23]. Inflammasome formation is triggered by numerous substances and has been shown to be activated after TBI in rodents and humans [24,25,26]. PAMPs and DAMPs are recognized by toll like receptors (TLR) initiating a cascade of events including TBI-induced cellular potassium efflux, increased intracellular calcium, subsequent mitochondrial dysfunction and excitotoxicity, which result in the formation and activation of the inflammasome [27]. The inflammasome activation involves cleavage of caspase-1 through the use of a scaffolding adaptor protein known as the apoptosis-associated speck-like protein containing a caspase recruiting domain (ASC), which oligomerizes to form an ASC speck. ASC specks bind to caspase-1, resulting in the formation of the inflammasome complex [28,29,30,31] and downstream activation of the pro-inflammatory cytokines interleukin (IL)-18 and IL-1β [32].

Biomarkers are specific proteins that are used as indicators of the status of different physiological processes in an individual [33,34,35,36,37,38,39]. Biomarkers are often used to determine the stage or severity of an underlying disease or injury [40,41,42,43,44,45,46]. TBI presents as a multi-factorial series of events that affect a variety of cells within the central nervous system (CNS) including neurons, microglia, astrocytes, oligodendrocytes and endothelial cells [47,48]. However, a variety of biomarkers need to be identified in order to gain a better understanding of the different molecular events that affect different cell types after TBI in the clinical setting. Two biomarkers have been thus far approved by the FDA for the monitoring of TBI patients. These biomarkers are ubiquitin carboxy-terminal hydrolase (UCH-L1) and glial fibrillary acidic protein (GFAP) [49,50,51,52,53,54,55]. UCH-L1 is expressed in neurons, and it is highly upregulated after TBI, whereas GFAP is expressed in astrocytes [56]. Thus, these two approved biomarkers for TBI offer clinicians an assessment of the degree of neuronal degradation and astroglial activation after brain injury. However, to date, no approved fluid biomarker or series of biomarkers is available to determine the inflammatory response in the acute setting after TBI.

Previous studies have shown that inflammasome proteins are potentially effective indicators of TBI severity and pathological outcomes in TBI [26]. Levels of ASC and caspase-1 are elevated in the blood of TBI patients with increased ASC levels correlating with more severe injury and worse outcomes [26]. Additionally, ASC and IL-18 were elevated in the cerebral spinal fluid (CSF) of patients after TBI [26], and caspase-1 was correlated with increased intracranial pressure and poor outcomes [57]. Moreover, several inflammatory cytokines have been described in the literature as potential biomarkers of TBI. For example, the elevation of tumor necrosis factor-α (TNF-α) [58,59,60], IL-8 [61,62,63] and IL-10 [58,61,64] have been reported in the CSF and serum of patients with TBI, and protein levels of IL-6 in plasma have been shown to correlate with brain injury severity [58,64,65].

In addition to TBI, we have previously shown that inflammasome proteins are reliable biomarkers of the inflammatory response in several conditions such as stroke [66], Alzheimer’s disease [67], multiple sclerosis [68], age-related macular degeneration [69], psoriasis [70] and non-alcoholic steatohepatitis [71], indicating that the inflammasome plays a major role in the pathophysiology of a variety of diseases affecting the CNS and the periphery. Moreover, those findings highlight the usefulness of inflammasome signaling proteins as biomarkers of injury and disease.

Despite ample evidence for the increased expression of a variety of inflammatory proteins in the CSF and blood of patients with TBI when compared to healthy uninjured controls, few studies have aimed to determine the biomarker characteristics of these inflammatory proteins, including the receiver operating characteristic (ROC) curve as well as the determination of cut-off points to identify the respective sensitivity and specificity of the different inflammatory biomarkers. In addition, previous studies have not compared the area under the curve (AUC) between different inflammatory biomarkers with the goal of identifying which inflammatory biomarkers are more suitable surrogates of the inflammatory response taking place acutely after TBI. Here, we measure the protein levels of inflammasome signaling proteins and inflammatory cytokines associated with TBI to then determine the biomarker characteristics of these proteins as well as the contribution of these inflammatory proteins to long term outcomes as determined by the Glasgow-Outcome Scale-Extended (GOS-E) and to injury severity as determined by the Glasgow-Comma Scale (GCS). Importantly, we follow a systematic approach to determine the suitability of each biomarker as a surrogate of inflammation following TBI and compare the AUC between each biomarker to identify which biomarkers have the potential to be more reliable biomarkers that can be used in the clinical setting.

## 2. Results

### 2.1. Inflammasome Proteins and Inflammatory Cytokines Are Elevated in TBI Patients

**Figure 1:**
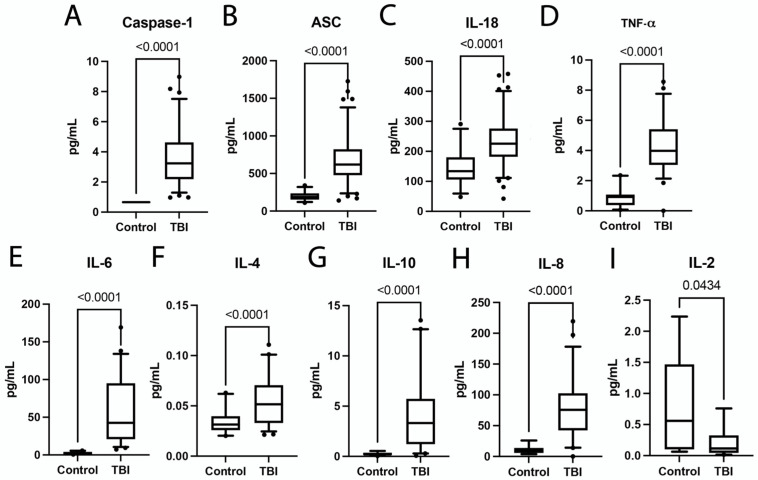
Inflammatory cytokines and Inflammasome Proteins are Elevated after TBI. Simple Plex Assay and MSD-VPLEX Inflammatory Panel of blood serum from TBI patients and age-matched controls. Data were analyzed utilizing a two-tailed Mann–Whitney nonparametric test. Inflammatory cytokines and inflammasome proteins that showed a statistically significant increase after TBI were plotted. Box and whisker plots show mean and quartiles for each inflammatory protein of interest with respective *p* values listed above. Dots correspond to data points outside the 5th and 95th percent confidence interval. Results showed that (**A**) Caspase-1: N: Control: 31, TBI: 78; (**B**) ASC: N: Control: 28, TBI: 91; (**C**) IL-18: N: Control: 31, TBI: 90; (**D**) TNF-α: N: Control: 21, TBI: 51; (**E**) IL-6: N: Control: 21, TBI: 46; and (**F**) IL-4: N: Control: 20, TBI: 50; (**G**) IL-10: N: Control: 19, TBI: 41; (**H**) IL-8: N: Control: 12, TBI: 52 were all significantly elevated in TBI patients when compared to controls. (**I**) IL-2: N: Control: 9, TBI: 19 was significantly decreased in TBI patients when compared to controls.

Increased inflammatory activity through inflammasome and cytokine signaling has been previously reported in animal and human TBI studies [27,72]. In order to determine which inflammatory proteins were elevated in this cohort of human TBI patients, we analyzed the levels of inflammasome proteins caspase-1 (Figure 1A), ASC (Figure 1B) and IL-18 (Figure 1C), and the inflammatory cytokines TNF-α (Figure 1D), IL-6 (Figure 1E), IL-4 (Figure 1F), IL-10 (Figure 1G), IL-8 (Figure 1H) and IL-2 (Figure 1I) in the blood serum of these patients and compared them to age-matched healthy controls. TBI patients had significantly elevated levels of inflammasome signaling proteins caspase-1 (Figure 1A), ASC (Figure 1B) and IL-18 (Figure 1C), as well as significantly elevated levels of cytokines TNF-α (Figure 1D), IL-6 (Figure 1E), IL-4 (Figure 1F), IL-10 (Figure 1G) and IL-8 (Figure 1H). In contrast, the levels of IL-2 were higher in the serum of healthy uninjured controls when compared to the serum of TBI patients (Figure 1I). Moreover, we found no significant difference in the levels of IL-12 when comparing the control with the TBI group (Figure S1). Therefore, these results indicate that TBI patients have sustained an acute increase in inflammatory activity after TBI.

### 2.2. Inflammatory Biomarkers of TBI

**Figure 2:**
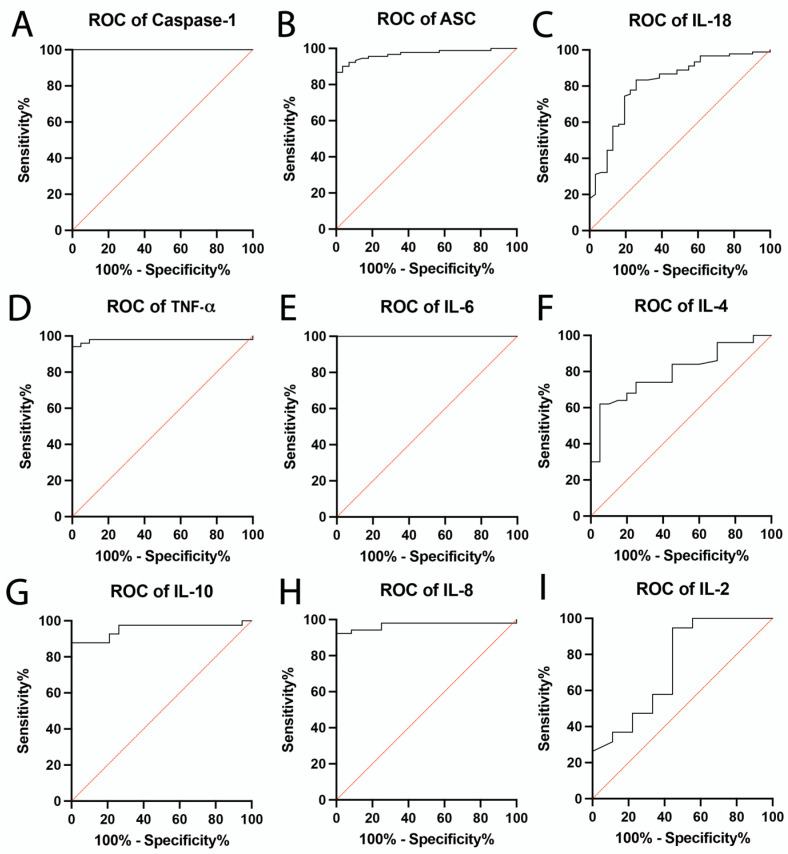
ROC of Inflammatory Biomarkers. ROC and AUC were calculated for each inflammatory cytokine and inflammasome proteins that were significantly different when comparing healthy uninjured controls and TBI patients. (**A**) Caspase-1: *N*: Control: 31, TBI: 78; (**B**) ASC: *N*: Control: 28, TBI: 91; (**C**) IL-18: *N*: Control: 31, TBI: 90; (**D**) TNF-α: *N*: Control: 21, TBI: 51; (**E**) IL-6: *N*: Control: 21, TBI: 46; and (**F**) IL-4: *N:* Control: 20, TBI: 50; (**G**) IL-10: *N:* Control: 19, TBI: 41; (**H**) IL-8: *N*: Control: 12, TBI: 52; (**I**) IL-2: *N*: Control: 9, TBI: 19.

Previous studies have shown that inflammasome proteins are potentially promising biomarkers for determining TBI pathological outcomes [24,26,57]. In order to determine the biomarker reliability of the inflammasome proteins caspase-1 (Figure 2A), ASC (Figure 2B) and IL-18 (Figure 2C), and the inflammatory cytokines TNF-α (Figure 2D), IL-6 (Figure 2E), IL-4 (Figure 2F), IL-10 (Figure 2G), IL-8 (Figure 2H) and IL-2 (Figure 2I) in the context of TBI, we plotted the ROC curve for each protein (Figure 2). Of the inflammatory cytokines examined, IL-6 (Figure 2E) had the highest AUC 1.0 (Table 1) with a sensitivity of 100% and a specificity of 100% (Table 2). TNF-α had an AUC of 0.98 AUC (96% sensitivity, 95% specificity), IL-10 and IL-8 also presented high AUC values (0.97 and 0.95, respectively), whereas IL-4 had an AUC of 0.79 (74% sensitivity, 75% specificity) and IL-2 an AUC of 0.74 (95% sensitivity, 56% specificity).

Of the inflammasome proteins examined, caspase-1 (Figure 2A) had the highest AUC at 1.0 (Table 1) with a sensitivity of 100% and a specificity of 100%, followed by ASC with an AUC of 0.97 with a sensitivity of 92% and a specificity of 93% (Table 2). IL-18 presented an AUC of 0.81 (sensitivity of 83%, specificity of 74%). These results indicate that caspase-1, ASC, IL-18, TNF-α, IL-6 IL-8 and IL-10 are reliable biomarkers of TBI with AUC values above 0.80.

### 2.3. Comparison between ROC Curves for Identified Inflammatory Biomarkers

**Figure 3:**
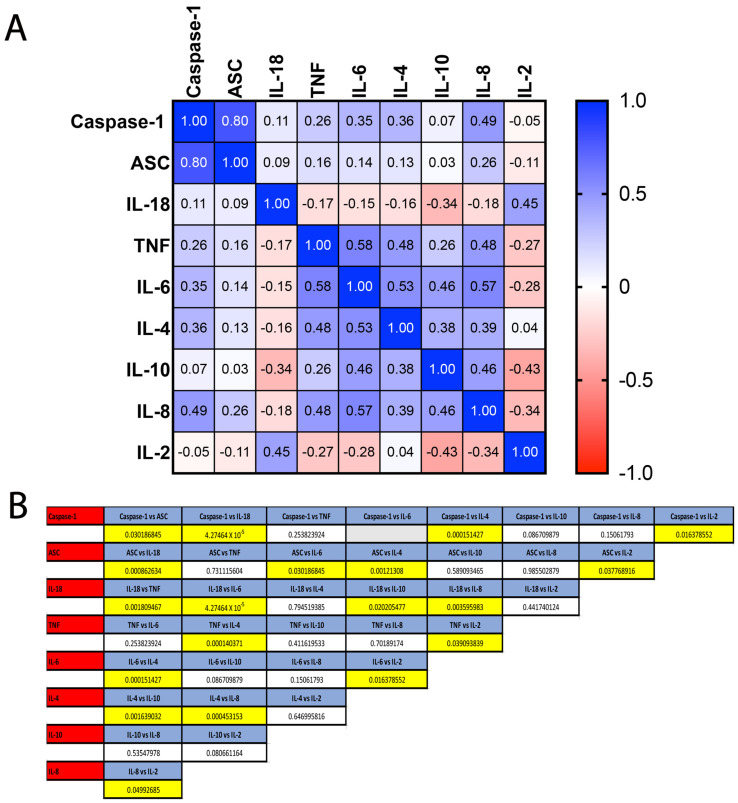
ROC Comparison among Inflammatory Biomarkers. (**A**) Correlation matrix using a Pearson correlation among inflammatory Biomarkers. (**B**) *p*-values of significance for the comparison among the inflammatory proteins analyzed: caspase-1, ASC, IL-18, TNF- α, IL-6, IL-4, IL-10 and IL-8.

To compare the ROC curves for caspase-1, ASC, IL-18, TNF-α, IL-6, IL-4, IL-10, IL-8 and IL-2, a Pearson correlation coefficient was first obtained from a correlation matrix (Figure 3A and Figure S2). The highest correlation was found between ASC and caspase-1 with a coefficient of correlation of 0.8, followed by a correlation of 0.58 between IL-6 and TNF-α, 0.57 between IL-6 and IL-8 and 0.53 between IL-6 and IL-4. After finding the coefficient of correlation, the ROC curves were compared according to the formula (Equation (1)):

(1) $$z=\frac{\left(A_{1}-A_{2}\right)}{\sqrt{SE_{1}{}^{2}+SE_{2}{}^{2}-2rSE_{1}SE_{2}}}.$$

ROC curve comparison analysis indicated that the ROC between caspase-1 and ASC (*p* = 0.03), caspase-1 and IL-18 (*p* = 4.27 × 10^−5^), caspase-1 and IL-4 (*p* = 0.0001) as well as caspase-1 and IL-2 (*p* = 0.01) were significantly different from each other (Figure 3B). Similarly, the ROC between ASC and other analytes differed from IL-18 (*p* = 0.0009), IL-6 (*p* = 0.03), IL-4 (*p* = 0.001) and IL-2 (*p* = 0.04). For IL-18, it also differed from TNF-α (*p* = 0.002), IL-6 (*p* = 4.27 × 10^−5^), IL-10 (*p* = 0.02) and IL-8 (*p* = 0.004). For TNF-α, the ROC curves also differed with IL-4 (*p* = 0.0001) and IL-2 (*p* = 0.04). For IL-6, the ROC curves differed with IL-4 (*p* = 0.0002) and IL-2 (*p* = 0.02). For IL-4, IL10 (*p* = 0.002) and IL-18 (*p* = 0.0005), and for IL-8 the ROC curve also differed to that of IL-2 (*p* = 0.05). Taken together, these analyses highlight caspase-1 and IL-6 as useful inflammatory biomarkers superior to all other biomarkers examined in this study; however, caspase-1 and IL-6 were not different from each other (Figure 3B). ASC and TNF-α were not different from each other but ASC was more reliable than IL-18 and IL-4. Similarly, IL-10 and IL-8 were not different from each other, and IL-8 was more reliable than IL-2.

### 2.4. Inflammatory Biomarkers of Injury Severity

**Figure 4:**
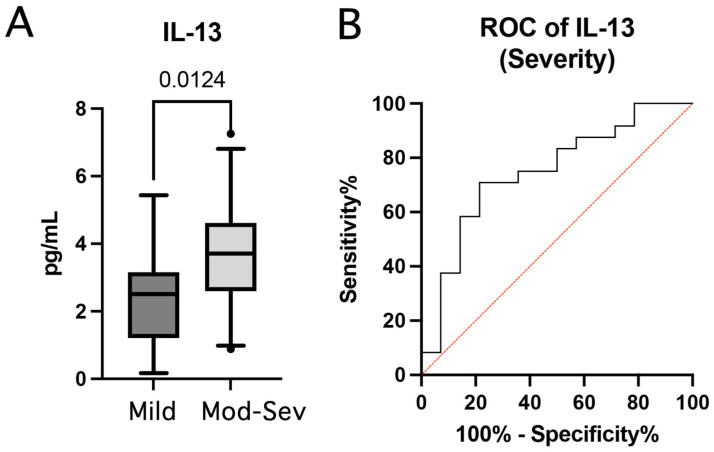
Inflammatory biomarkers as predictors of TBI severity. (**A**) Box and whisker plots showing the protein levels in pg/mL of IL-13 as a biomarker of injury severity. Box and whiskers are shown for the 5th and 95th percentile. Dots correspond to data points outside the 5th and 95th percent confidence interval. (**B**) ROC curves indicate the AUC (sensitivity vs. 1-specificity) for IL-13. Mild: *N* = 14, Moderate to severe: *N* = 24.

Next, we screened inflammatory biomarkers to determine whether there was a difference in the levels of these proteins between patients that presented mild TBI and those who had moderate to severe TBI as determined by the GCS. Patients with mild TBI were those who presented a GCS between 13 and 15; whereas patients with a GCS between 3 and 12 were grouped in the moderate to severe cohort. Of all the analytes measured in this study, IL-13 was the only protein to be elevated in the moderate to severe group when compared to patients in the mild TBI group (Figure 4A). Moreover, we calculated the ROC curve for IL-13 and found that the AUC for IL-13 was 0.75 (Figure 4B) with a 95% confidence interval between 0.5815 to 0.9126 and an SEM of 0.085 (*p* = 0.01), indicating that IL-13 discriminates between mild and moderate to severe TBI. Furthermore, with a cut-off point of 3.12 pg/mL, the sensitivity and specificity of IL-13 were 71% and 79%, respectively. This resulted in a PPV of 85% and a NPV of 61% with an accuracy of 74%.

We further fit a multivariate linear regression model using a stepwise approach to predict inflammatory biomarkers that contribute to the GCS. Accordingly, using as predictors the inflammatory proteins caspase-1, ASC, IL-18, TNF-α, IL-2, IL-4, IL-6, IL-8, IL-10, IL-12, IL-13 and IFN-γ.

The best model was chosen based on the AIC (14.37) by the stepwise method, and then the estimate (coefficients), standard error and *p*-values for each predictor and intercept (slope), as well as the BIC (35.52), residuals (Figure S3A), RMSE (1.65), mean of residuals (−6.25 × 10^−17^), confidence intervals, and the DW autocorrelation test for the best fit model, were calculated. An adjusted R^2^ value (0.78) was obtained for the model to determine the approximate contribution of the fitted model to the GCS (Table 3). Thus, based on this model, we determined that the GCS score is contributed to in part by IL-12, IL-13 and the log (IL-12), considering an adjusted R^2^ of 0.78 and a *p*-value of 0.03, consistent with our findings of IL-13 as a biomarker of injury severity after TBI based on the GCS.

### 2.5. Inflammatory Biomarkers of Outcome

**Figure 5:**
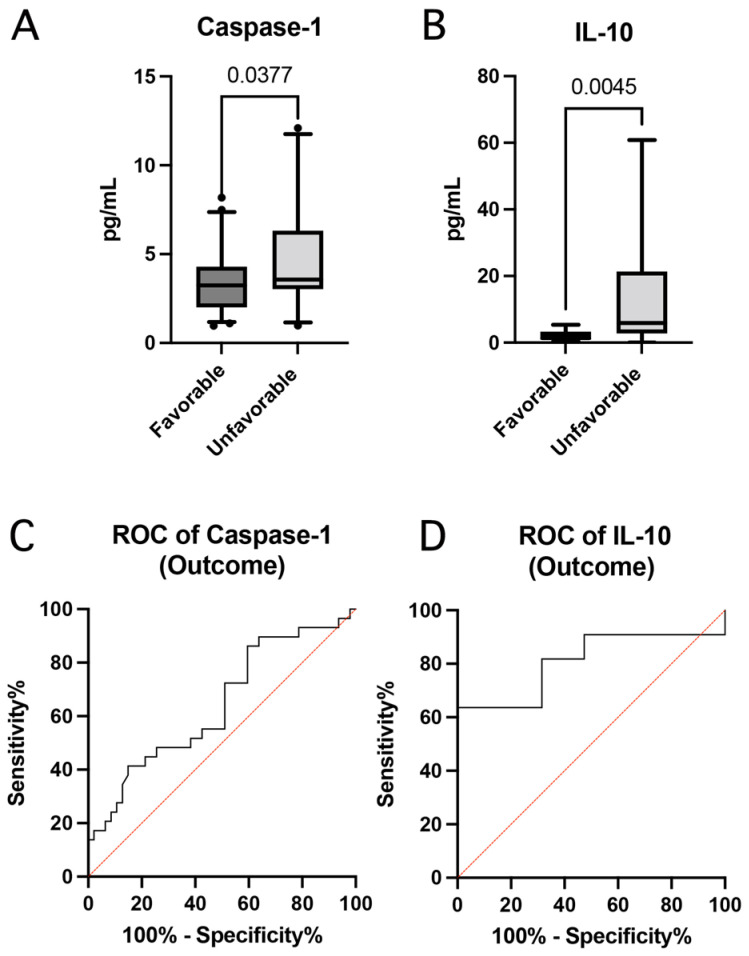
Inflammatory biomarkers as predictors of TBI outcome. Box and whisker plots showing the protein levels in pg/mL of caspasea-1 (**A**) and IL-10 (**B**) as a biomarker of outcome after TBI. Box and whiskers are shown for the 5th and 95th percentile. Dots correspond to data points outside the 5th and 95th percent confidence interval. ROC curves indicate the AUC (sensitivity vs. 1-specificity) for (**C**) caspase-1: Favorable: *N* = 47, Unfavorable: *N* = 29 and (**D**) IL-10: Favorable: *N* = 19, Unfavorable: *N* = 11.

To determine the contribution of inflammatory proteins to outcomes according to the GOS-E, we first divided the outcomes as favorable and unfavorable and then determined if there was a statistically significant difference between the levels of the inflammatory proteins caspase-1, ASC, IL-18, TNF-α, IL-2, IL-4, IL-6, IL-8, IL-10, IL-12, IL-13 and IFN-γ with regard to favorable (GOSE scores of 5–8) vs. unfavorable (GOSE scores of 1–4) outcomes. Of the protein analyzed, caspase-1 (Figure 5A) and IL-10 (Figure 5B) were significantly elevated in patients that presented unfavorable outcomes.

Following the identification of caspase-1 and IL-10 as proteins that were elevated in patients with unfavorable outcomes, we aimed to identify whether these two analytes are good biomarkers of outcomes in TBI patients. The ROC curves of caspase-1 (Figure 5C) and IL-10 (Figure 5D) were 0.64 (*p* = 0.03) and 0.81 (*p* = 0.006), respectively. The SEM for the ROC of caspase-1 was 0.07 with a 95% confidence interval between 0.5127 and 0.7720. For IL-10, the SEM was 0.097 with a 95% confidence interval between 0.6182 and 0.9990. Moreover, with a cut-off point of 3.33 pg/mL, and a likelihood ratio of 1.3, the sensitivity and specificity of caspase-1 were 55% and 57%, respectively, with a PPV of 68% and an NPV of 44% with an accuracy of 56%. In contrast, for IL-10, with a cut-off point of 5.55 pg/mL, the sensitivity and specificity were 64% and 100%, respectively, with a PPV of 100% and an NPV of 61% with an accuracy of 77%.

We then fitted a multivariate linear regression model using caspase-1 and IL-10 as the predictors to explain the GOS-E. After analyzing residuals (Figure S3B) and adding a logarithmic transformation for caspase-1 levels, the adjusted R^2^ value (0.65) was obtained for the model to determine the approximate contribution of IL-10 and caspase-1 to the GOS-E (*p* = 0.03) (Table 4).

## 3. Discussion

Recent advancements in biomarker analytical technology have provided for the greater sensitivity and consistency across assays, thereby allowing the identification of substances in serum as potential biomarkers. In this study, we used two technologies: electrochemiluminescence (MESO QuickPlex SQ120, MSD, Rockville, MD, USA) and microfluidics (Ella, Protein Simple, San Jose, CA, USA) to measure inflammatory biomarkers in the serum of patients with TBI. Overall, we demonstrate that caspase-1, ASC, IL-6 and TNF were the most reliable pro-inflammatory biomarkers of the acute response after TBI, whereas IL-10 was the best anti-inflammatory biomarker. Therefore, the serum of TBI patients is an ideal source for the measurement of the signaling proteins that may be used for diagnostic and prognostic potential to estimate the degree of neuronal damage, astrocyte activation, and the inflammatory response involving microglial neutrophils and other inflammatory cells in TBI patients.

Previous studies have measured a variety of inflammatory proteins in healthy individuals and TBI patients [73]. Many of these studies did not evaluate the actual biomarker characteristics of these proteins. However, it is not sufficient to solely measure levels of proteins in control and TBI groups, but it is critical to calculate the ROC curve and to obtain the AUC by plotting the sensitivity in the *y*-axis and 1-specificity in the *x*-axis for each analyte. In addition, cut-off points should be identified with their respective sensitivity and specificity. AUC values between 0.9 and 1.0 correspond to an excellent biomarker; from 0.8 to 0.9, a good biomarker; from 0.7 to 0.8, a fair biomarker; from 0.6 to 0.7, poor and from 0.5 to 0.6, a failed analyte [74]. Our data show that caspase-1 and IL-6 with an AUC of 1.0 were the best inflammatory biomarkers of those examined in this study, followed by TNF-α and ASC with an AUC of 0.98 and 0.97, respectively, and IL-8 and IL-10 with an AUC of 0.97 and 0.96, respectively. Furthermore, the most sensitive inflammatory biomarkers were caspase-1 and IL-6, followed by TNF-α, IL-2, ASC and IL-8, whereas the most specific biomarkers were caspase-1, IL-6, IL-8. IL-10, TNF-α ASC. Moreover, caspase-1 and IL-6 presented an accuracy of 100%, followed by TNF-α with 96%, IL-8, 94% and ASC and IL-10 with an accuracy of 92%. Taken together, these data indicate that caspase-1, ASC, TNF-α, IL-6, IL-10 and IL-8 are the most reliable diagnostic inflammatory biomarkers of TBI, among those studied. However, when there are several biomarkers that have similar AUC values, it is important to determine if the ROC curves differ among the different biomarkers. Thus, following the determination of the ROC curves for each biomarker, we then compared the ROC for each of them, and identified significant differences between different biomarkers despite many of them having high AUC values. For instance, we found significant differences between caspase-1 and ASC. However, the ROC for caspase-1 and IL-6 were not significantly different, indicating that both of these analytes have the same biomarker potential based on their respective biomarker characteristics, yet this is not to say that each of these biomarkers does not provide different information pertaining to the acute inflammatory response after TBI. Similarly, the ROC curve for ASC and TNF- were not found to be significantly different either, and the AUC of IL-6 was found to be superior to that of ASC, consistent with the significance found between caspase-1 and ASC. Overall, the findings of this study highlight the importance of caspase-1, ASC, IL-6 and TNF as pro-inflammatory biomarkers of the acute response after TBI, whereas IL-10 was the best anti-inflammatory biomarker.

Additionally, in this study, we dichotomized the GOS-E into favorable and unfavorable outcomes to identify whether inflammatory proteins were significantly different between patients with different outcomes after TBI. We found that caspase-1 and IL-10 were elevated in patients with unfavorable outcomes when compared to those with favorable outcomes. IL-10 is secreted by numerous cells of the CNS after injury and has been shown to play a protective role by reducing cytokine activity, proinflammatory activity, and apoptosis [75,76]. Additionally, IL-10 inhibits IL-2 activity, and has been shown in numerous studies to be increased after stroke or TBI [75,76]. Although IL-10 has a pro-survival purpose, increased IL-10 expression after injury has been associated with worsened pathological outcomes with higher expression associated with increased chance for mortality [75]. Our results support these observations and further suggest an interplay between pro-inflammatory (caspase-1) and anti-inflammatory (IL-10) proteins in the pathogenesis that play a role in long-term outcomes in patients with TBI. Biomarker analysis indicated that the AUC for caspase-1 was 0.64 and the AUC for IL-10 was 0.81, suggesting that IL-10 was a more reliable biomarker of long-term outcomes with a cut-off point of 5.55 pg/mL. Previous studies have shown that serum levels of TNF-α remain elevated for at least one-year post-injury [15,77]. Serum levels of IL-6 have been shown to be more elevated in more severe cases of TBI, and that elevated IL-6 levels were associated with worsened outcomes [77]. Similarly, IL-8 increased IL-8 expression in serum or CSF of TBI patients is associated with an increased chance for mortality and overall worsened pathological outcomes [78], and may be attributed to the chemoattractant properties of IL-8 that recruit and activate monocytes to the site of injury, increasing the overall inflammatory response after TBI or stroke [76,78].

Lastly, we found that IL-13 was elevated in patients with moderate to severe TBI as determined by the GCS. The exact role of IL-13 in CNS injury pathology is still up for debate [78,79], although some studies have suggested that IL-13 plays a neuroprotective role in that it reduces inflammatory activity, reduces axonal loss, and mediates microglia polarization, encouraging the adoption of the anti-inflammatory phenotype [76]. IL-13 treatment improves pathological outcomes in a murine model of TBI [79]. Furthermore, IL-2 has been shown to be decreased after TBI [80], and IL-13 has been shown to have shared functionality with IL-4, and synergizes with IL-2 to promote IFN production [76]. Our findings indicate that IL-13 had an AUC of 0.75 and a cut-off point of 3.12 pg/mL, with a sensitivity of 71% and a specificity of 79%; thus, IL-13 is a fair biomarker of TBI injury severity. Moreover, a multivariate linear regression model consisting of IL13, IL-2 and IL-12 indicated that, combined, these three biomarkers contribute to the GCS with an adjusted R^2^ of 0.78, thus highlighting the importance of IL-13 and a key biomarker of injury severity.

## 4. Materials and Methods

### 4.1. Participants

Study specimens from TBI patients were acquired from Son Espases University Hospital (Palma de Mallorca, Spain). The study was approved by the Comité Ético de las Islas Baleares (IRB protocol number 3127/15). Written informed consent was obtained from a family member or proxy according to the IRB (Table 5). Healthy age-matched controls were acquired from BiolVT (Hicksville, NY, USA). Informed consent was obtained from specimen donors. Control samples were obtained by donors participating in the study Prospective Collection of Samples for Research funded by SeraTrials, LLC. with IRB number 20170439. Blood samples from TBI patients used in this study were collected in the range of approximately 60 to 720 min after TBI with a median of 367.5 min (~6 h after TBI). Exclusion criteria consisted of patients with normal findings on the CT scan on admission, patients with a major extracranial trauma (defined as extracranial Injury Severity Score > 18 points), and patients with past medical history relevant to CNS pathology such as brain tumor, meningitis, cerebral vasculitis or stroke.

### 4.2. Data Collection

Patients’ clinical data were recorded and reviewed using the electronical medical records from the hospital (Power Chart; Millenium, 2011, Cerner Corporation, Kansas City, MO, USA). We collected all the variables included in the International Mission for Prognosis and Analysis of Clinical Trials in TBI (IMPACT) prognostic calculator for each patient. We also collected the GCS that first responders wrote in their prehospital report or the hospital admission GCS if the former was not available. The 6-month outcome was assessed using the extended version of the Glasgow Outcome Scale (GOSE) by a trained Neurosurgery Intensive Care Unit attending (JRP) by telephone consultation, and he was blinded to biomarker analysis.

### 4.3. Simple Plex Assay

The serum concentrations of inflammasome proteins (Caspase-1, ASC and IL-18) were measured in 93 TBI patients and in 31 age-matched controls via Ella System (Protein Simple) as described in [69]. Briefly, samples were loaded as 50 μL of diluted sample into sample wells of a CART with 1 mL of washing buffer loaded separately into respective buffer wells. An assay was run using the Runner Software (version 3.5.2.20, San Jose, CA, USA). Samples were then automatically analyzed utilizing the Simple Plex Explorer (version 3.7.2.0, San Jose, CA, USA) [70].

### 4.4. MSD V-PLEX Inflammatory Panel

Serum levels of the inflammatory cytokines TNF-α, IL-2, IL-4, IL-6, IL-8, IL-10, IL-12p-70, IL-13 and interferon (IFN)-γ were measured utilizing the V-PLEX Proinflammatory Panel 1 (MSD) as in [67]. All relevant controls, detection antibodies, standards, reagents, and dilutants were supplied by the manufacturer and prepared in accordance with manufacturer’s instructions. Briefly, samples were diluted 2-fold prior to loading into the plate. Plate wells were washed three times with wash buffer prior to sample loading. Then, 50 μL of sample was loaded into respective plate wells and allowed to incubate for 2 h at room temperature on a plate shaker. After incubation, plate wells were washed three times with wash buffer. Detection antibody was then added to plate wells and was allowed to incubate for 2 h at room temperature on a plate shaker. After antibody incubation, plate wells were washed three times with wash buffer. A 2X Read buffer was then added to each well and the plate was analyzed utilizing the MESO QuickPlex SQ120 (MSD, Rockville, MD, USA) and DISCOVERY WORKBENCH software (version 4.0.12, Rockville, MD, USA).

### 4.5. Statistical & Biomarker Analysis

Simple Plex and V-PLEX data from TBI and control samples were analyzed utilizing Prism 9 software (GraphPad). Outliers were removed prior to further statistical analyses using the Robust regression and **Out**lier removal (ROUT) method with a Q set to 1%. Descriptive statistics were run, and normality was determined by the Shapiro–Wilk test or the D’Agostino and Pearson Test. Non-parametric data were analyzed using a two-tailed Mann-Whitney test and parametric data were analyzed using a two-tailed *t*-test. *p*-value of significance was set to *p* < 0.05.

Receiver operating characteristics (ROC) were calculated to obtain the area under the curve (AUC) in order to obtain cut-off points and the respective specificity, sensitivity and likelihood ratio. The cut-off point for each analyte was chosen based on the highest likelihood ratio in the sensitivity vs. 1-specificity plot, favoring a higher sensitivity than specificity values, to obtain assays with a higher likelihood of reliability for each analyte [81]. Positive and negative predictive values were also calculated along with overall assay accuracy.

A comparison of ROC curves between inflammatory biomarkers was carried out as described in [82] using the Equation (1) to obtain a critical ratio *z*:

The *p*-value was determined using the following formula using Microsoft Excel (version 16.57, Redmond, WA, USA):

= 2 × (1 − NORMSDIST(z)).

A Pearson correlation was carried out to obtain r in order to calculate the z-score to allow for a comparison of ROC curves between analytes obtained from the same samples.

Linear regression analyses to explain the GCS and the GOS-E were fit using all the inflammatory proteins analyzed in this study through a stepwise approach based on the lowest Akaike information criterion (AIC) using RStudio/RMarkdown (Version 1.2.5033, Boston, MA, USA) and were then fitted to obtain the estimate, standard error and *p*-values for each predictor and the intercept. The Bayesian information criterion (BIC), residuals, root mean-square error (RMSE), mean of residuals, confidence intervals, and the autocorrelation using the Durbin–Watson (DW) statistic were then calculated for the best fit model. The Durbin–Watson (DW) statistic was used to test for autocorrelation. After identifying a best fit model, data points underwent logarithmic transformation to normalize the distribution of the data. An adjusted r-squared value was obtained to determine the approximate contribution of these three proteins to either the GCS or the GOS-E. The final models were then further evaluated by residual analysis with and without logarithmic transformation.

## 5. Conclusions

In conclusion, we provide a systematic approach for inflammatory biomarker identification that includes: (1) measurement of the levels of inflammatory problems in the serum of affected and unaffected individuals to determine if there are statistical differences between groups; (2) determination of the diagnostic biomarker characteristics (AUC, sensitivity, specificity, likelihood ratio, accuracy, PPV and NPV) of each inflammatory protein or analyte that was statistically significant when comparing the levels between affected and unaffected individuals; (3) comparison of the ROC among the different biomarkers to identify potential biomarker differences between groups; (4) dichotomization of the GCS into mild and moderate to severe outcomes to determine if there are inflammatory biomarkers that meet the criteria as useful biomarkers of injury severity; and (5) dichotomization of the GOS-E into favorable and unfavorable outcomes to determine if there are inflammatory biomarkers that meet the criteria as useful biomarkers of long-term outcomes. Taken together, we identified the caspase-1, ASC, IL-18, TNF-α, IL-2, IL-4, IL-6, IL-8, IL-10 and IL-12 as surrogate biomarkers in serum of the inflammatory response acutely after TBI. Thus, the use of inflammatory biomarkers when combined with GFAP and UCH-L1 may offer clinicians a better understanding of the overall scope of injury and provide a probable prognosis and potential for disability considering a variety of mechanisms contributing to the TBI pathology, including neuronal damage (UCH-L1), reactive astrogliosis (GFAP) and inflammation (caspase-1, ASC, TNF-α and IL-6). Identification of clinically relevant biomarkers of the inflammatory response after TBI allow for future studies looking at how therapeutics affect these biomarkers, and how the effects of those therapeutics on biomarkers affect injury severity and functional outcomes in patients after TBI. In addition, the identification of these inflammatory biomarkers provides the opportunity of developing therapeutics that can be used to more specifically treat the inflammatory response associated with TBI. Taken together, with modern approaches for the measurement of biomarkers with higher accuracy and sensitivity than in the past, and with the identification of biomarkers of neuronal damage, reactive astrogliosis, and inflammation, personalized care for TBI patients is becoming a more tangible reality. Furthermore, in light of the results in this project, future studies in clinically relevant animal models of TBI should focus on understanding the individual and synergistic effects of therapeutically targeting the inflammatory proteins identified as relevant biomarkers of the inflammatory response after TBI for their ability to improve histopathological and functional outcomes.

## Figures and Tables

**Table 1:** ROC Analysis.

Biomarker	Area	Std. Error	95% C.I.	*p*-Value
Caspase-1	1.0	0	1.0 to 1.0	<0.0001
ASC	0.97	0.01384	0.9428 to 0.9971	<0.0001
IL-18	0.8143	0.04538	0.7254 to 0.9033	<0.0001
TNF-α	0.9776	0.01963	0.9391 to 1.000	<0.0001
IL-6	1.0	0	1.0 to 1.0	<0.0001
IL-4	0.7945	0.05424	0.6882 to 0.9008	0.0001
IL-10	0.9538	0.02697	0.9009 to 1.0	<0.0001
IL-8	0.9696	0.2115	0.9281 to 1.0	<0.0001
IL-2	0.7398	0.1084	0.5274 to 0.9522	0.0437
IL-12	0.5333	0.07812	0.3802 to 0.6864	0.6645
IL-13	0.5126	0.1093	0.2985 to 0.7267	0.8858

**Table 2:** Cut-off point in serum of TBI patients.

Biomarker	Cut-Off Point (pg/mL)	Sensitivity (%)	Specificity (%)	LR	PPV (%)	NPV (%)	Accuracy (%)
Caspase-1	>0.8150	100	100		100	100	100
ASC	>284	92	93	12.92	98	79	92
IL-18	>156	83	74	3.229	90	61	81
TNF-α	>2.202	96	95	20.18	98	91	96
IL-6	>6.443	100	100		100	100	100
IL-4	>0.03868	74	75	2.96	88	54	74
IL-10	>0.6527	88	100		100	79	92
IL-8	>29.18	92	100		100	75	94
IL-2	<0.5145	95	56	2.132	82	83	82
IL-12	<158.1	60	57	1.40	75	40	59
IL-13	>2.271	76	57	1.784	87	40	72

**Table 3:** Linear Regression Model to predict GCS.

GCS	Estimate	Std. Error	*p*-Value	Confidence Interval
Intercept	21.245347	2.221427	0.000668	15.07767794 to 27.413016974
IL-13	−1.519195	0.332622	0.010282	−2.44270084 to −0.595688402
IL-12	−0.016000	0.004802	0.029063	−0.02933364 to −0.002666043
LOG(IL-2)	2.095605	0.670269	0.035304	0.23463864 to 3.956571427
Adjusted R^2^		0.7798		
BIC		35.51754		
RMSE		1.644861		
Mean of Residuals		−6.25 × 10^−17^		
DW Statistic				
rho ! = 0	*p*-value = 0.812			
rho < 0	*p*-value = 0.443			
rho > 0	*p*-value = 0.561			

GCS = 21.25 − 1.52 (IL-13) − 0.02 (IL-12) + 2.10 × LOG(IL-12).

**Table 4:** Linear Regression Model to predict GOS-E.

GCS	Estimate	Std. Error	*p*-Value	Confidence Interval
Intercept	9.1238	0.7938	8.74 × 10^−05^	7.0832670 to 11.1643303
IL-10	0.2483	0.1416	0.1398	−0.1156099 to 0.6122821
LOG(Caspase-1)	−2.5997	0.6700	0.0116	−4.3220676 to −0.8772845
Adjusted R^2^		0.6524		
BIC		24.35273		
RMSE		0.8338086		
Mean of Residuals		1.39 × 10^−17^		
DW Statistic				
rho ! = 0	*p*-value = 0.574			
rho < 0	*p*-value = 0.716			
rho > 0	*p*-value = 0.316			

GOS-E = 9.12 + 0.25(IL-10) − 2.60 × LOG(caspase-1).

**Table 5:** Summary of demographic data and clinical characteristics in patients with TBI.

	TBI (*N* = 93)
Gender (*n*, %)	
Male	74 (80%)
Female	19 (20%)
Age (years) median (Range)	47 (15–83)
Injury Mechanism (*n*; %)	
Fall	52 (56%)
Assault	5 (5%)
MVA	36 (39%)
Glasgow Coma Scale (*n*; %)	
3–8	37 (40%)
9–12	20 (21%)
13–15	34 (37)
Undetermined	2 (2%)
Motor score (*n*; %)	
M6	38 (41%)
M5	29 (32%)
M4	4 (4%)
M3	2 (2%)
M2	1 (1%)
M1	14 (15%)
Undetermined	5 (5%)
Pupillary Reactivity (*n*; %)	
Both reactive	78 (84%)
1 reactive	8 (9%)
None reactive	7 (7%)
Hospital length of stay (days) median (Range)	13 (1–149)
ICU length of stay (days) median (Range)	5 (1–90)

TBI: Traumatic Brain injury; MVA: motor vehicle accident; ICU: Intensive Care Unit.

## Data Availability

Data is contained within the article and supplementary material.

## Supplementary Materials

The following supporting information can be downloaded at: https://www.mdpi.com/article/10.3390/ph15060660/s1, Figure S1: IL-12 profile in serum. Figure S2: (A) Autocorrelation Plot of all analytes examined in this study; (B) *p*-values for the respective comparisons shown in the autocorrelation plot. Figure S3: Residual analysis of the multivariate linear regression models used to explain GCS (A) and GOS-E (B) were performed to determine the goodness of fit of each of the models.
