# Supplementary material for: Inflammatory Biomarkers of Traumatic Brain Injury
Source: Pharmaceuticals (Basel). 2022 May 25;15(6):660. doi: 10.3390/ph15060660 (PMC9227014; doi:10.3390/ph15060660)
Supplement: Supplementary file 1 [file pharmaceuticals-15-00660-s001.zip › pharmaceuticals-1724143-supplementary.pdf]

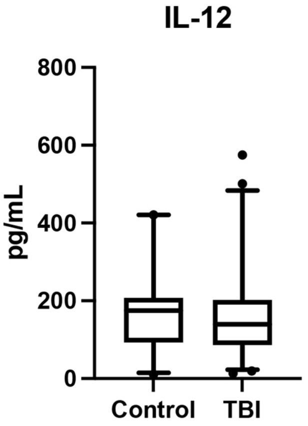

Figure S1: IL-12 profile in serum. MSD-VPLEX Inflammatory Panel of blood serum from TBI patients and age matched controls. Box and whisker plots show mean and quartiles. Dots correspond to data points outside the 5th and 95th percent confidence interval. N: Control: 21, TBI: 45.

A

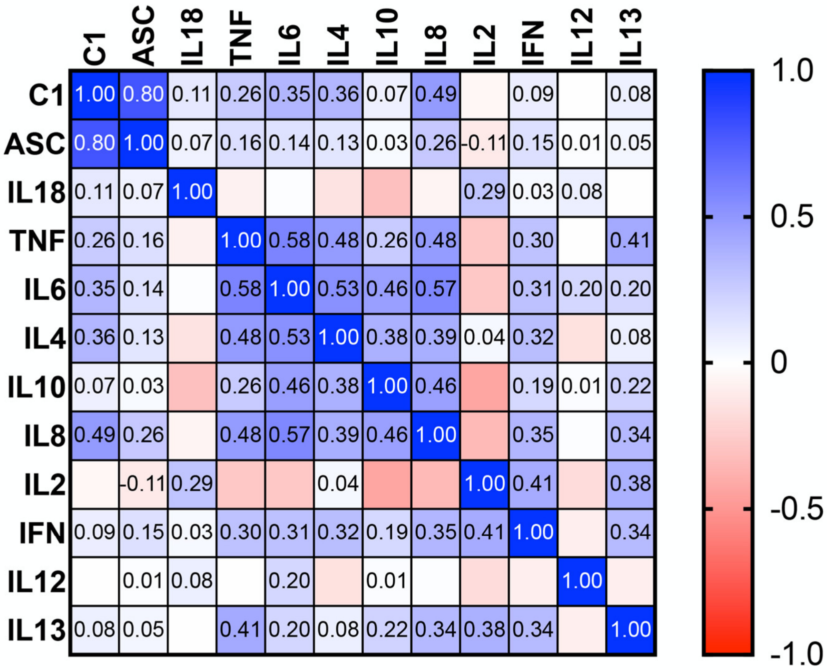

B

	C1	ASC	IL18	TNF	IL6	IL4	IL10	IL8	IL2	IFN	IL12	IL13
C1		3.5622E-18	0.32982871	0.0998328	0.03612023	0.02237335	0.68528936	0.00111715	0.86286364	0.59338027	0.97444652	0.63253127
ASC	3.5622E-18		0.51551282	0.26245559	0.36109853	0.38158001	0.87336045	0.06172133	0.65663176	0.30799447	0.97379687	0.74003808
IL18	0.32982871	0.51551282		0.60557679	0.99339096	0.31374174	0.05240022	0.64516625	0.24052364	0.81680661	0.60140303	0.93715334
TNF	0.0998328	0.26245559	0.60557679		3.3821E-05	0.0007089	0.12418387	0.00053454	0.31209903	0.04148679	0.97182768	0.00354021
IL6	0.03612023	0.36109853	0.99339096	3.3821E-05		0.00014695	0.00512444	3.484E-05	0.281497	0.04816858	0.23774145	0.19809887
IL4	0.02237335	0.38158001	0.31374174	0.0007089	0.00014695		0.0188068	0.00550784	0.88686426	0.03296423	0.34999492	0.57102429
IL10	0.68528936	0.87336045	0.05240022	0.12418387	0.00512444	0.0188068		0.00322949	0.10725454	0.27311673	0.94522064	0.18451955
IL8	0.00111715	0.06172133	0.64516625	0.00053454	3.484E-05	0.00550784	0.00322949		0.17295088	0.01650078	0.97991616	0.01724811
IL2	0.86286364	0.65663176	0.24052364	0.31209903	0.281497	0.88686426	0.10725454	0.17295088		0.13382645	0.53791612	0.13266718
IFN	0.59338027	0.30799447	0.81680661	0.04148679	0.04816858	0.03296423	0.27311673	0.01650078	0.13382645		0.60541039	0.02571528
IL12	0.97444652	0.97379687	0.60140303	0.97182768	0.23774145	0.34999492	0.94522064	0.97991616	0.53791612	0.60541039		0.59214281
IL13	0.63253127	0.74003808	0.93715334	0.00354021	0.19809887	0.57102429	0.18451955	0.01724811	0.13266718	0.02571528	0.59214281	

Figure S2: A) Autocorrelation Plot of all analytes examined in this study; **B)** p-values for the respective comparisons shown in the autocorrelation plot.

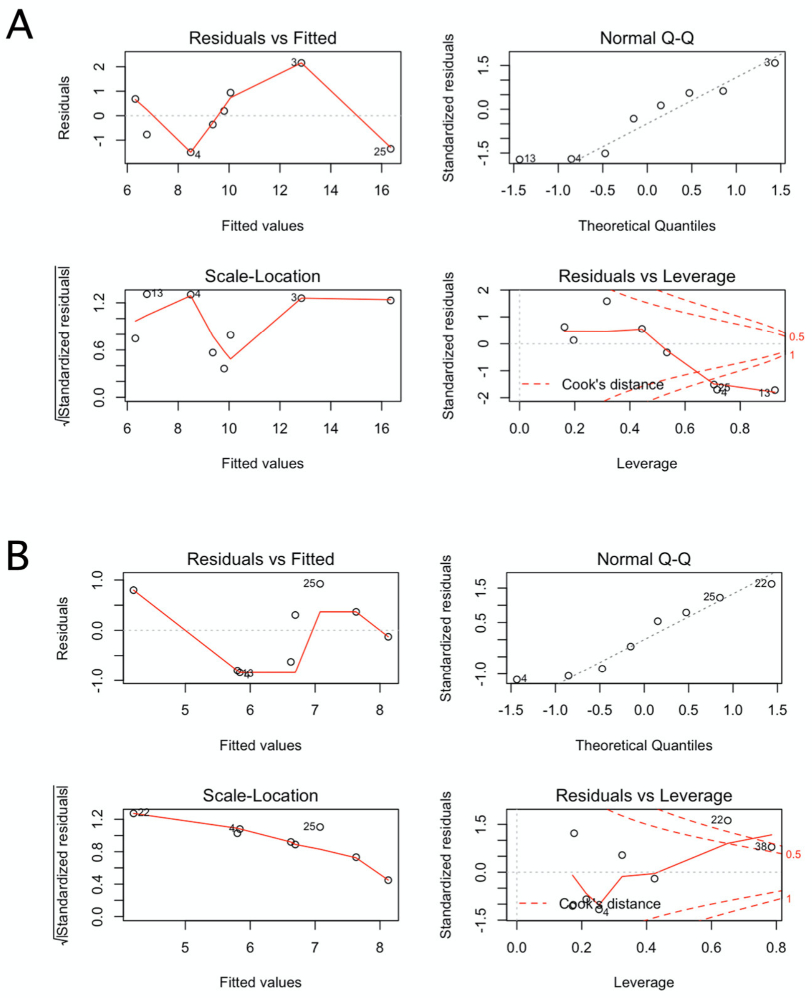

Figure S3: Residual Analysis. Residual analysis of the multivariate linear regression models used to explain GCS (**A**) and GOS-E (**B**) were performed to determine the goodness of fit of each of the models.
